# High Expression of the Long Noncoding RNA SNHG15 in Cancer Tissue Samples Predicts an Unfavorable Prognosis of Cancer Patients: A Meta-Analysis

**DOI:** 10.1155/2020/3417036

**Published:** 2020-07-15

**Authors:** Cheng Zhang, Yang Ke, Xin Liu, Xinghong Wang, Yuehua Li, Jian Zhou, Heng Zhang, Lin Wang

**Affiliations:** ^1^Department of Hepatobiliary Surgery, The Second Affiliated Hospital of Kunming Medical University, Kunming 650101, China; ^2^Department of Hepatobiliary Surgery, The Sixth People's Hospital of Chengdu, Chengdu 610041, China; ^3^Department of Dermatology, The Second Affiliated Hospital of Chengdu Medical College, Chengdu 610051, China; ^4^Department of Hepatobiliary Surgery, The First People's Hospital of Yunnan Province, Kunming 650032, China

## Abstract

**Background:**

Although the prognostic value of lncRNA small nucleolar RNA host gene 15 (SNHG15) expression in cancers has been evaluated in many studies, the results remain controversial. This meta-analysis aimed to clarify the role of SNHG15 in the prognosis of different cancer patients.

**Materials and Methods:**

Eligible studies were selected from PubMed, PMC, EMBASE, Web of Science, and Cochrane Library according to the inclusion and exclusion criteria (up to December 20, 2019). The primary outcome was overall survival (OS) and recurrence-free survival (RFS). The secondary outcome was other clinicopathological parameters (including advanced TNM stage, lymph node metastasis, distant metastases, and gender). The Cancer Genome Atlas (TCGA) dataset was used to verify the analysis results.

**Results:**

Eleven eligible studies were eventually included, involving 9 different types of cancer and 1,079 patients. The high expression of SNHG15 was indicative of a significantly poor OS of cancer patients (HR = 1.96, 95% CI = 1.55–2.47, *P* < 0.00001). Subgroup analysis showed that the high expression of SNHG15 was associated with a significantly poor OS of patients with digestive cancer (HR = 1.91, 95% CI = 1.38–2.66, *P*=0.0001), but not lung cancer (HR = 1.83, 95% CI = 0.89–3.76, *P*=0.010). The RFS of patients with high expression of SNHG15 was shorter than that of patients with low expression of SNHG15 (HR = 2.03, 95% CI = 1.46–2.83, *P* < 0.00001). In addition, high SNHG15 expression level was significantly correlated with later TNM stage (OR = 3.05, 95% CI = 2.31–4.02, *P* < 0.00001), lymphatic metastasis (OR = 3.20, 95% CI = 2.30–4.45, *P* < 0.00001), and distant metastasis (OR = 5.05, 95% CI = 2.15–11.85, *P*=0.0002). The TCGA verification results were consistent with those observed in our meta-analysis.

**Conclusion:**

High expression of the long noncoding RNA SNHG15 in cancer tissue samples predicts an unfavorable prognosis for cancer patients. LncRNA SNHG15 can be used as an adverse prognostic biomarker for cancer patients.

## 1. Introduction

Malignant tumors are among the leading causes of morbidity and mortality for people of all ages worldwide [1, 2]. Despite the considerable progress in cancer treatments such as immunotherapy and targeted therapy, the survival rate for most cancers is still low [3, 4]. Finding potential biomarkers of cancer prognosis and elucidating their role and mechanisms will greatly impact the care of cancer patients.

Long noncoding RNAs (lncRNAs) are transcripts with a length of more than 200 nucleotides and are widely found in nucleus, cytoplasm, and exosome [5]. They usually cannot be translated into specific proteins because they do not have functional open reading frames [6]. Over the past decade, many studies have reported the critical role of such transcripts in physiological processes, like embryogenesis, immune cell activation, blood cell maturation, and cell-to-cell communication [5, 7–9]. Moreover, many lncRNAs are dysregulated in human cancers, and lncRNAs may act as oncogenes or tumor suppressors in tumorigenesis and metastasis processes [5, 10, 11]. They have been proven to be a novel and promising prognostic biomarker and treatment targets in human cancer [12, 13].

Small nucleolar RNA host gene 15 (SNHG15) is a newly discovered LncRNA located on chromosome 7p13 [14]. SNHG15 is reported to be significantly upregulated in a variety of cancer tissues compared with adjacent noncancer tissues [15–17], and abnormal overexpression of SNHG15 in cancer samples is associated with poor prognosis and high risk of metastasis in different cancers, including breast, colorectal, gastric, and prostate cancers [15, 18–28]. However, the prognostic value of SNHG15 expression in thyroid cancers and glioma is controversial [29, 30]. Therefore, we meta-analyzed all relevant publications and TCGA database to achieve a comprehensive understanding of the relationship between SNHG15 expression and cancer patient prognosis and evaluated whether SNHG15 could be a potential biomarker for the prognosis of cancer patients.

## 2. Materials and Methods

### 2.1. Literature Search Strategies

Article search was performed in the databases, including PubMed, PMC, EMBASE, Web of Science, and Cochrane Library. The time limit for searching was from the construction of these databases to December 20, 2019. The terms used for the search were as follows: (small nucleolar RNA host gene 15 OR SNHG15) AND (cancer OR carcinoma OR tumor OR neoplasm OR malignant). References of the literature included were also traced back.

### 2.2. Inclusion and Exclusion Criteria

The inclusion criteria for research studies were as follows: (1) research conducted on any type of human cancer; (2) measuring the expression of SNHG15 in tumor tissue; (3) investigating the relationship between SNHG15 expression level and prognosis or clinicopathological characteristics; and (4) reporting the hazard ratio (HR) or odds ratio (OR) with 95% confidence interval (CI), or with sufficient data available for calculation.

The exclusion criteria were as follows: (1) duplicate articles; (2) editorials, letters, expert opinions, case reports, and reviews.

The primary outcome was overall survival (OS) and recurrence-free survival (RFS). The secondary outcome was other clinicopathological characteristics (including TNM stage, lymph node metastasis, distant metastases, gender, and tumor size).

### 2.3. Data Collection

Two researchers independently (Cheng Zhang and Yang Ke) extracted data from each original publication, and a third researcher (Xin Liu) resolved the differences. The extracted information included name of the first author, year of publication, country, type of cancer, sample size, the detection method for lncRNA, internal control, outcome index, and cutoff value. For studies reporting only Kaplan–Meier curves, Engauge Digitizer (version 12.1) software was used to extract survival data [31] and the HRs and 95% CIs were calculated using the EXCEL program file provided by Tierney et al. [32]. The quality of the included studies was assessed using the Newcastle–Ottawa Scale (NOS) criteria, including selection (4 points) and comparability (2 points); studies with NOS scores above 6 were considered of high quality.

### 2.4. Bioinformatics Analysis and Verification

Gene Expression Profiling Interactive Analysis (GEPIA) database (http://gepia.cancer-pku.cn/) was used to analyze the differential expression of SNHG15 between cancer and normal tissues and the correlation of SNHG15 expression with OS of different cancer types.

### 2.5. Statistical Analysis

Statistical analysis was performed using RevMan 5.3.3 software and Stata SE 15.1 software. HR for death or recurrence and 95% CI were calculated between the high and low SNHG15 expression group. Patients with HR  ≥ 1 indicated a poor prognosis. Furthermore, the correlation between SNHG15 expression and clinicopathological characteristics was evaluated using OR and 95% CI. Heterogeneity between the included studies was determined by the *I*^2^ value from the Cochrane Q test and the *P* value from the chi-square test. If there was heterogeneity (*I*^2^ ≥ 50% or *P* < 0.05), the results were summarized using a random-effects model. Instead, fixed-effects models were used for analysis. Otherwise, a random-effects model was applied. Begg's funnel plot and Egger's test were used to assess publication bias. A sensitivity analysis was applied to assess the stability of the results. *P* ≤ 0.05 was considered statistically significant.

## 3. Results

### 3.1. Characteristics of Included Studies

**Figure 1:**
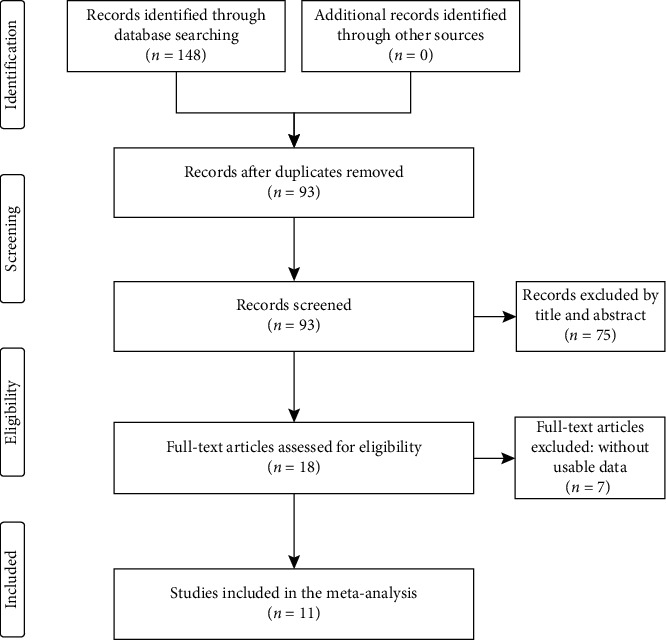
Flow diagram of the study search and selection.

As shown in Figure 1, 59 articles were initially detected according to the search strategy, and 41 articles remained after removing duplicates. After carefully screening the titles and abstracts based on the inclusion and exclusion criteria, 26 articles were excluded, including reviews, conference abstracts, and unrelated studies. After reading the full text of the remaining 15 articles, 4 articles were excluded due to incomplete data. Finally, 11 studies involving 1,078 patients were included in the meta-analysis [15, 18, 19, 21–28].

All the 11 studies included were from China, with 9 different types of cancer, viz., hepatocellular carcinoma (HCC) [18], gastric cancer (GC) [19], colorectal cancer (CRC) [27], pancreatic cancer (PC) [21], non-small-cell lung cancer (NSCLC) [24–26], renal cell cancer (RCC) [22], thyroid cancer (TC) [23], breast cancer (BC) [25], and epithelial ovarian cancer (OC) [28]. All the included studies were tested for the expression of SNHG15 by quantitative reverse transcription PCR (qRT-PCR), but the cutoff value and the internal reference were different (Table 1). HR and 95% CI could be directly extracted in 2 studies [19, 23] and were calculated from the survival curve in the remaining 9 studies using the Engauge Digitizer software [31, 32]. The characteristics of the included studies are summarized in Table 1.

### 3.2. Association between SNHG15 Expression and OS

**Figure 2:**
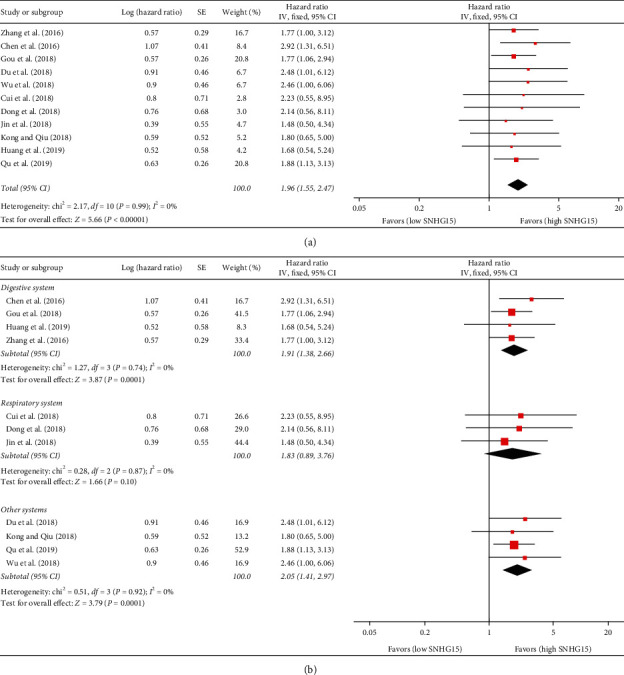
Forest plot for the association between SNHG15 expression and OS in (a) all cancer patients and (b) subgroup analysis based on different cancer types.

The association between SNHG15 expression and OS was reported in the 11 studies [15, 18, 19, 21–28]. As there was no statistical heterogeneity among the 11 studies (*P*=0.99, *I*^2^ = 0%), a fixed-effects model was chosen to estimate the combined HR and 95% CI. The results showed that the HR for death between the high SNHG15 expression group and the low SNHG15 expression group was 1.96 (95% CI = 1.55–2.47, *P* < 0.00001; see Table 2 and Figure 2(a)).

**Figure 3:**
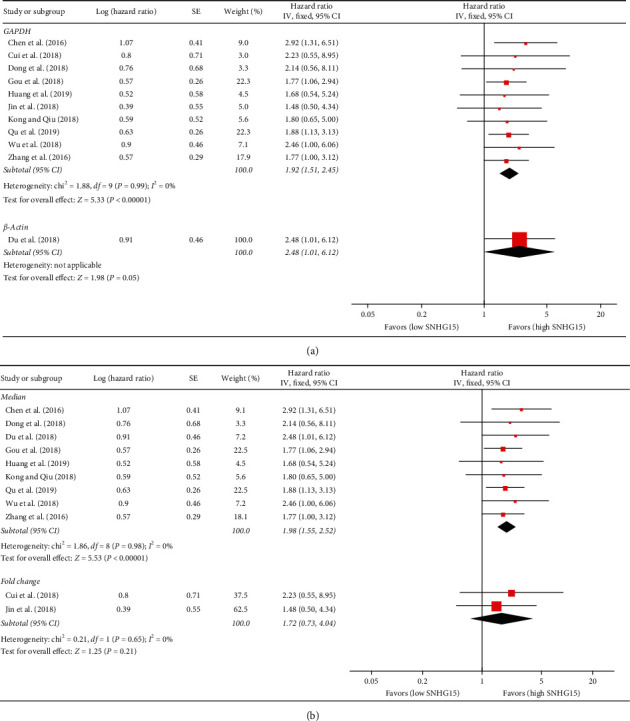
Forest plot for the association between SNHG15 expression and OS in a subgroup analysis based on (a) internal control and (b) cutoff.

Given that the 11 studies reported 9 different types of cancer, we divided these studies into three subgroups, including digestive system (including HCC, CRC, GC, and PC) [18, 19, 21, 27], respiratory system (including NSCLC) [24–26], and other systems (including RCC, TC, BC, and OC) [15, 22, 23, 28]. Since no significant heterogeneity between different subgroup studies was detected (*I*^2^ = 0%, *P*=0.74 for digestive system, *I*^2^ = 0%, *P*=0.87 for respiratory system, and *I*^2^ = 0%, *P*=0.92 for other system), we used a fixed-effects model to complete the synthesis of the data. The results of the subgroup analysis showed that the HR and 95% CI for death between the high SNHG15 expression group and the low SNHG15 expression group in digestive, respiratory, and other cancers were 1.91 (95% CI = 1.38–2.66, *P*=0.0001), 1.83 (95% CI = 0.89–3.76, *P*=0.10), and 2.05 (95% CI = 1.41–2.97, *P*=0.0001; see Table 2 and Figure 2(b)). Furthermore, we also performed a series of subgroup analyses based on cutoff values and internal controls. The results showed that when GAPDH or *β*-actin was used as an internal control, or median value was used as a cutoff test, high expression of SNHG15 was significantly associated with a shorter OS (see Table 2, Figures 3(a) and 3(b)).

### 3.3. Association between SNHG15 Expression and RFS

**Figure 4:**
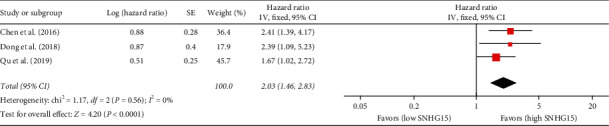
Forest plot for association between SNHG15 expression and RFS.

Three studies reported the association between SNHG15 expression and RFS [19, 25, 28]. As there was no statistical heterogeneity among the 3 studies (*I*^2^ = 0%, *P*=0.56), a fixed-effects model was used. The combined HR for RFS between the high SNHG15 expression group and the low SNHG15 expression group was 2.03 (95% CI = 1.46–2.83, *P* < 0.00001; see Figure 4), suggesting that an increase in SNHG15 expression was significantly associated with a shorter OS.

### 3.4. Association between SNHG15 Expression and Other Clinicopathological Parameters

**Figure 5:**
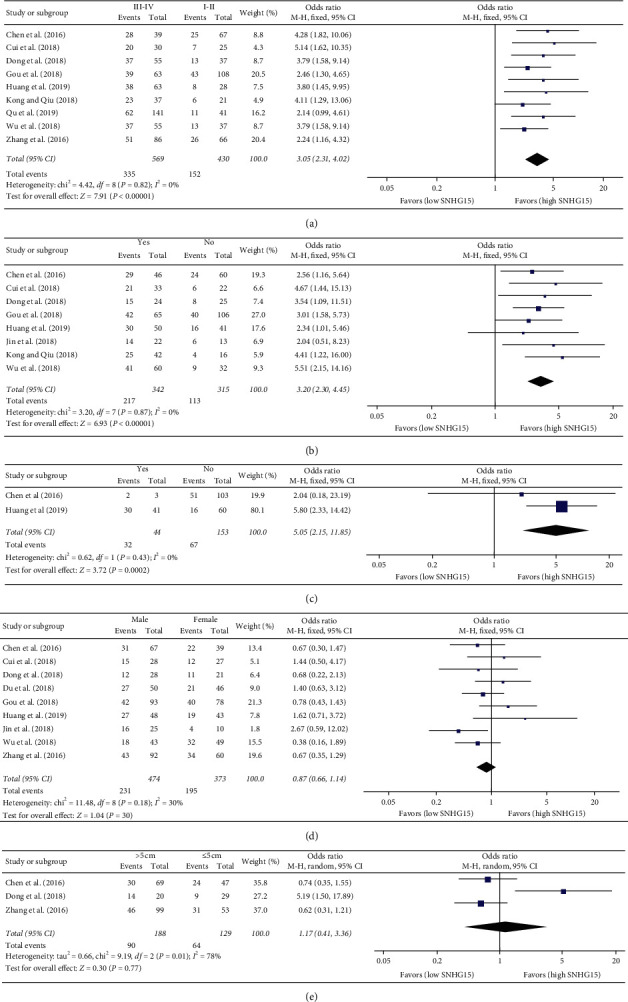
Forest plots for the association between SNHG15 expression and (a) TNM stage (III-IV vs. I-II), (b) lymph node metastasis (yes vs. no), (c) distant metastasis (yes vs. no), (d) gender (male vs. female), and (e) tumor size (>5 cm vs. ≤5 cm).

As shown in Table 3, high SNHG15 expression was associated with advanced TNM stage (OR = 3.05, 95% CI = 2.31–4.02, *P* < 0.00001; see Table 3 and Figure 5(a)), lymph node metastasis (OR = 3.20, 95% CI = 2.30–4.45, *P* < 0.00001; see Table 3 and Figure 5(b)), and distant metastases (OR = 5.05, 95% CI = 2.15–11.85, *P*=0.0002; see Table 3 and Figure 5(c)). In contrast, no statistical correlation was observed between SNHG15 expression and gender (*P*=0.30; see Table 3 and Figure 5(d)) and tumor size (*P*=0.77; see Table 3 and Figure 5(e)). Additionally, due to insufficient age-related data on tumor differentiation in the included studies, no meta-analysis was performed on the association between SNHG15 expression and these clinical-pathological parameters.

### 3.5. Sensitivity Analysis and Publication Bias

**Figure 6:**
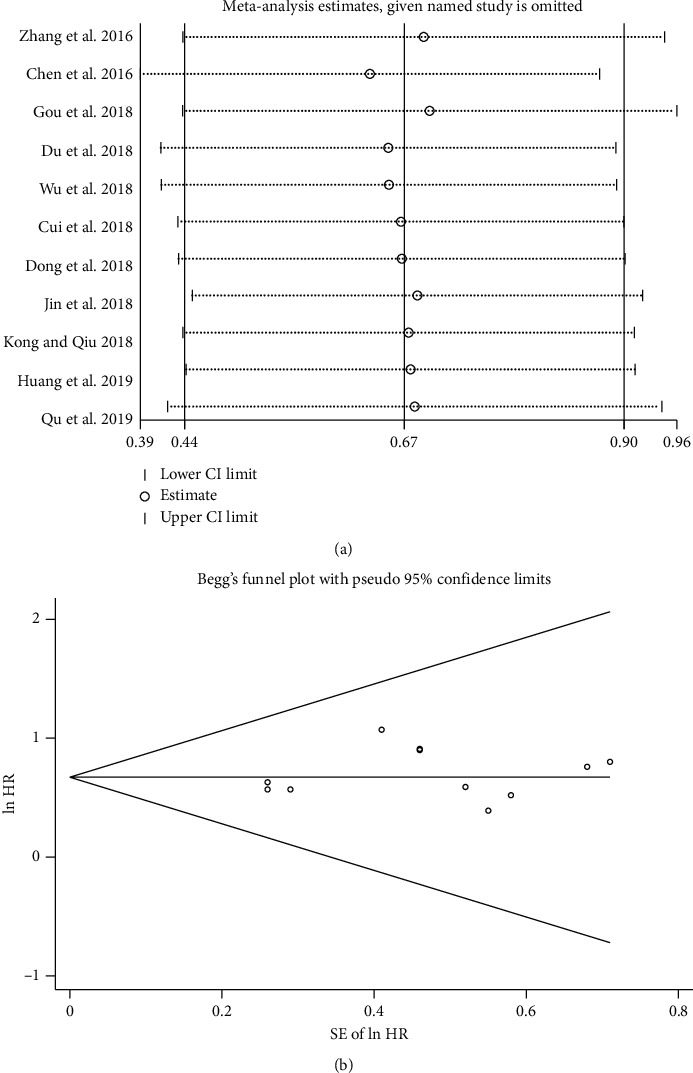
Sensitivity analysis and publication bias. (a) Sensitivity analysis of the association between SNHG15 expression and OS. (b) Begg's funnel plot of publication bias for OS.

As shown in Figure 6(a), the deletion of any included studies had no significant impact on the results, suggesting that our results were reasonable and reliable.

Furthermore, we performed Begg's funnel plot and Egger's test to evaluate the publication bias of the correlation between SNHG15 expression and OS. The Begg funnel plot is shown in Figure 6(b), and the results of the Egger test was *P*=0.409. The results indicated no significant publication bias in the studies included in this meta-analysis.

The sensitivity analysis and Begg's test between SNHG15 expression and RFS were also performed, and the results suggested that our results on RFS were reasonable and reliable (see Figures S1(a) and S1(b)).

### 3.6. Verification Results in the TCGA Dataset

**Figure 7:**
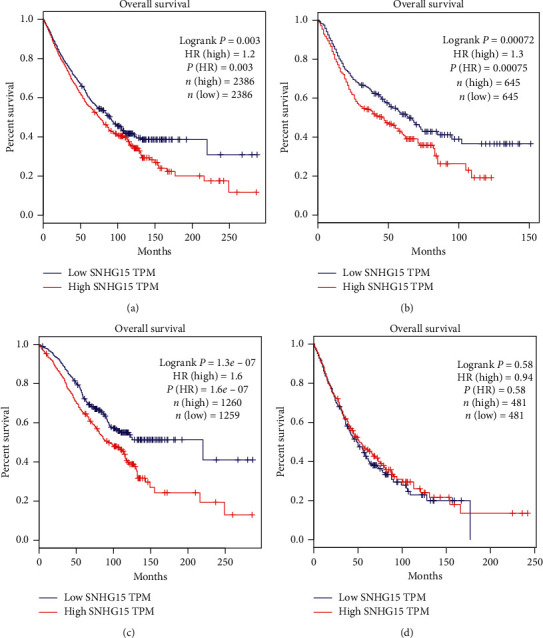
Validation of SNHG15 in the TCGA dataset. (a) Survival curves of SNHG15 are plotted for 9 kinds of cancers involved in this meta-analysis from the TCGA dataset. (b) Survival curves of SNHG15 are plotted for digestive cancers, including hepatocellular carcinoma (HCC), colorectal cancer (CRC), gastric cancer (GC), and pancreatic cancer (PC), from the TCGA dataset. (c) Survival curves of SNHG15 are plotted for other cancers, including renal cell cancer (RCC), thyroid cancer (TC), breast cancer (BC), and epithelial ovarian cancer (OC), from the TCGA dataset. (d) Survival curves of SNHG15 are plotted for non-small-cell lung cancer, including lung adenocarcinoma (LUAD) and lung squamous cell carcinoma (LUSC), from the TCGA dataset.

We evaluated the correlation between SNHG15 expression and OS in the 9 malignant tumors involved in this meta-analysis through the TCGA dataset. According to the median expression of SNHG15 in each of the 9 malignant tumors, patients with an expression level higher than the median were considered in the high-expression group, and patients with an expression level lower than the median were considered in the low-expression group. As shown in Figure 7(a), the cancer patient population with high SNHG15 expression levels had a significantly poorer OS than those with low SNHG15 expression levels (HR = 1.2, *P*=0.012), which was consistent with our meta-analysis results. We also evaluated the correlation between SNHG15 expression and OS in digestive (including HCC, CRC, GC, and PC), respiratory (including lung adenocarcinoma (LUAD) and lung squamous cell carcinoma (LUSC)) and in other (including RCC, TC, BC, and OC) malignant tumors in TCGA. The result showed that patients with high SNHG15 expression levels had significantly poorer OS than those with low SNHG15 expression levels in digestive cancers (HR = 1.3, *P*=0.00072; see Figure 7(b)) and other cancers (HR = 1.6, *P*=1.3 × 10^−7^; see Figure 7(c)). However, there was no correlation between SNHG15 expression and OS in respiratory cancers (HR = 0.94, *P*=0.58; see Figure 7(d)). In addition, we evaluated the correlation between SNHG15 expression and other clinicopathological parameters in the 9 malignant tumors through the TCGA dataset. Consistent with our meta-analysis results, high SNHG15 expression was associated with advanced TNM stage (*P*=0.014; see Table 4), lymph node metastasis (*P* < 0.001; Table 4), and distant metastases (*P* < 0.001; see Table 4), while no significant correlation was observed between SNHG15 expression and gender (*P*=0.637; see Table 4).

## 4. Discussion

This study provides for the first time a systematic analysis of the relationship between SNHG15 expression and prognosis of patients with different types of cancer. The most important finding in this meta-analysis is that high expression of the long noncoding RNA SNHG15 in cancer tissues predicts an unfavorable prognosis for cancer patients. SNHG15 is one of the newly discovered popular lncRNAs involved in the development and progression of many malignant tumors. SNHG15 was first identified as a lncRNA with a short half-life in a cell stress response study [33]. Since then, a large number of studies have reported that SNHG15 is highly expressed in various malignant tumors, including GC, BC, CRC, HCC, NSCLC, TC, and OV [15, 18, 23, 25, 27, 28]. Furthermore, the high expression of SNHG15 is associated with poor prognosis of patients with different types of cancer [18, 22, 25].

Some molecular mechanisms were revealed by which SNHG15 acts as an oncogene. In the nucleus, SNHG15 can interact with the zinc finger domain of Slug, inhibiting Slug ubiquitination, and then the redundant Slug proteins inhibit E-cadherin transcription, promote the epithelial–mesenchymal transition (EMT), and stimulate the invasion and metastasis of the colon, breast, and renal cancer cells [22, 34]. In pancreatic cancer, SNHG15 can also bind to the enhancer of zeste homolog 2 and inhibit the expression of p15 and the Kruppel-like factor 2 by zeste homolog 2-mediated H3 lysine 27 trimethylation modification and finally promote the proliferation of pancreatic cancer cells [20]. In the cytoplasm, SNHG15 can also act as the competing endogenous RNA and competitively bind to a variety of microRNAs, influence the expression of multiple invasion related proteins, and thereby promote the proliferation, invasion, and migration of malignant tumor cells [35]. For example, in osteosarcoma, SNHG15 can directly bind miR-141 as a “molecular sponge” to promote osteosarcoma expression and cancer growth [36].

Different cancers display distinct clinical characteristics and biological behaviors. To reduce the heterogeneity between studies, a subgroup study was performed based on pathological classification. The results suggested that the increase of SNHG15 expression in the digestive subgroup (including HCC, CRC, GC, and PC) and RCC, TC, BC, and OC subgroup was significantly associated with a shorter OS, which suggested that SNHG15 may be a reliable prognostic biomarker for cancers of the digestive, urinary, thyroid, and reproductive organs. However, there was no significant correlation detected between SNHG15 and NSCLC.

Chemotherapy is a main treatment approach for various cancers. Previous studies have found that SNHG15 expression contributes to cisplatin resistance in BC [37] and temozolomide resistance in glioma [38]. In the present study, we found that SNHG15 expression correlated with lymphatic metastasis, distant metastasis, and later TNM stages, suggesting that (1) increased expression of SNHG15 may be closely related to the advanced characteristics of cancer; (2) clarification of the relationships between SNHG15 expression and clinical parameters may allow the identification of the patient population who can potentially benefit from chemotherapy.

Our study included a large number of samples, which confers a high statistical power. Therefore, the results of the present study are more stable and accurate than those of previous individual studies. However, some limitations should be noted in this meta-analysis. First, all studies were from China, so we used the TCGA database, which includes cancer cases from multiple regions including African American, American native, and Caucasian, to verify our meta-analysis results. Indeed, the bioinformatics results support our meta-analysis. A large sample size with multiple races is needed to confirm our findings in the future. Second, given the fact that individual patient data were not analyzed, certain data on the relationships between clinical parameters and SNHG15 could not be combined.

## 5. Conclusions

In conclusion, this is the first meta-analysis reporting that LncRNA SNHG15 can be used as a prognostic biomarker for cancer patients, especially cancers in the digestive, urinary, thyroid, and reproductive systems. In addition, the high expression level of SNHG15 was closely related to the advanced characteristics of cancer, indicating that patients without these advanced characteristics may be prime candidates for chemotherapy. More high-quality and large-sample studies are required to further confirm the prognostic role of SNHG15 in cancer.

## Figures and Tables

**Table 1:** Characteristics of the studies included in the meta-analysis.

Study	Year	Country	Cancer type	Detection method	Internal control	Cutoff	Total sample	Expression		Tumor stage	Metastasis analysis	Outcome	HR estimation	NOS score
								High	Low					
Zhang et al. [18]	2016	China	HCC	qRT-PCR	GAPDH	Median	152	77	75	I–IV	N/A	OS	SC	8
Chen et al. [19]	2016	China	GC	qRT-PCR	GAPDH	Median	106	53	53	I–IV	DM/LNM	OS/RFS	Reported	7
Gou et al. [21]	2018	China	PC	qRT-PCR	GAPDH	Median	171	82	89	I–IV	LNM	OS	SC	8
Du et al. [22]	2018	China	RCC	qRT-PCR	*β*-Actin	Median	96	47	52	T1–T4	N/A	OS	SC	7
Wu et al. [23]	2018	China	TC	qRT-PCR	GAPDH	Median	92	50	42	I–IV	LNM	OS	Reported	9
Cui et al. [24]	2018	China	NSCLC	qRT-PCR	GAPDH	FC	55	28	27	I–IV	LNM	OS	SC	7
Dong et al. [25]	2018	China	NSCLC	qRT-PCR	GAPDH	Median	49	23	26	I–IV	LNM	OS/RFS	SC	7
Jin et al. [26]	2018	China	NSCLC	qRT-PCR	GAPDH	FC	35	20	15	I–III	LNM	OS	SC	8
Kong and Qiu [15]	2018	China	BC	qRT-PCR	GAPDH	Median	58	29	29	I–IV	LNM	OS	SC	7
Huang et al. [27]	2019	China	CRC	qRT-PCR	GAPDH	Median	91	46	45	I–IV	DM/LNM	OS	SC	8
Qu et al. [28]	2019	China	OC	qRT-PCR	GAPDH	Median	182	73	109	I–IV	N/A	OS/RFS	SC	8

HCC: hepatocellular carcinoma; GC: gastric cancer; PC: pancreatic ductal cancer; RCC: renal cell carcinoma; TC: thyroid carcinoma; NSCLC, non-small-cell lung cancer; BC: breast cancer; CRC: colorectal cancer; EOC: epithelial ovarian cancer; qRT-PCR: quantitative reverse transcription PCR; GAPDH: glyceraldehyde-3-phosphate dehydrogenase; FC: fold change; LNM: lymph node metastasis; DM: distant metastasis; N/A: not available; OS: overall survival; SC: survival curve; RFS: recurrence -free survival; HR: hazard ratio; and NOS: Newcastle–Ottawa Scale.

**Table 2:** Subgroup meta-analysis of HRs for death.

Categories	Study number [references]	Total sample	*P* value	HR (95% CI) for OS	Heterogeneity		Model
					*I* ^2^ (%)	*P*_h_	
OS	11 [15, 18, 19, 21–28]	1087	<0.00001	1.96 (1.55–2.47)	0	0.99	Fixed
Cancer type							
Digestive system	4 [18, 19, 21, 27]	520	0.0001	1.91 (1.38–2.66)	0	0.74	Fixed
Respiratory system	3 [24–26]	139	0.1	1.83 (0.89–3.76)	0	0.87	Fixed
Other systems	4 [15, 22, 23, 28]	428	0.0001	2.05 (1.41–2.97)	0	0.92	Fixed
Internal control							
GAPDH	10 [15, 18, 19, 21, 23–28]	991	<0.00001	1.92 (1.51–2.45)	0	0.99	Fixed
*β*-Actin	1 [22]	96	0.0479	2.48 (1.01–6.12)	N/A	N/A	N/A
Cutoff							
Median	9 [15, 18, 19, 21–23, 25, 27, 28]	997	<0.00001	1.98 (1.55–2.52)	0	0.98	Fixed
Fold change	2 [24, 26]	90	0.21	1.72 (0.73–4.04)	0	0.65	Fixed

OS: overall survival; GAPDH: glyceraldehyde-3-phosphate dehydrogenase; HR: hazard ratio; and N/A: not applicable.

**Table 3:** Meta-analysis of the association between SNHG15 expression and clinicopathological parameters.

Clinicopathological parameters	Study number [references]	Total sample	*P* value	OR (95% CI) for OS	Heterogeneity		Model
					*I* ^2^ (%)	*P*_h_	
Gender (male vs. female)	9 [18, 19, 21–27]	847	0.30	0.87 (0.66–1.14)	30	0.18	Fixed
Tumor size (>5 cm vs. ≤5 cm)	3 [18, 19, 25]	317	0.77	1.17 (0.41–3.36)	78	0.01	Random
TNM stage (III-IV vs. I-II)	9 [15, 18, 19, 22–25, 27]	999	<0.00001	3.05 (2.31–4.02)	0	0.82	Fixed
Lymph node metastasis (yes vs. no)	8 [15, 19, 21, 23–27]	657	<0.00001	3.20 (2.30–4.45)	0	0.87	Fixed
Distant migration (yes vs. no)	2 [19, 27]	197	0.0002	5.05 (2.15–11.85)	0	0.43	Fixed

OR: odds ratio; OS: overall survival; and CI: confidence interval.

**Table 4:** Correlation between SNHG15 expression and other clinicopathological parameters in TCGA.

Clinicopathological parameters	Number of cases (*n* = 3430)	SNHG15 expression		*P* value
		High (*n* = 1787)	Low (*n* = 1643)	
Gender				0.637
Male	1734	896	838	
Female	1696	891	805	
TNM stage				0.014
III-IV	1090	602	488	
I-II	2340	1185	1155	
Lymph node metastasis				<0.001
Yes	1593	996	597	
No	1837	791	1046	
Distant migration				<0.001
Yes	215	161	54	
No	3215	1626	1589	

Median expression level was used as the cutoff. For analysis of the correlation between SNHG15 levels and clinical features, Pearson's chi-square tests were used. Results were considered statistically significant at *P* < 0.05.

## Data Availability

The data used to support the findings of this study are available from the corresponding author upon request.

## Supplementary Materials

Supplementary Materials Figure S1: sensitivity analysis and publication bias. (a) Sensitivity analysis of the association between SNHG15 expression and association and recurrence-free survival (RFS). (b) Begg's funnel plot of publication bias for RFS.
