# Supplementary material for: High Expression of the Long Noncoding RNA SNHG15 in Cancer Tissue Samples Predicts an Unfavorable Prognosis of Cancer Patients: A Meta-Analysis
Source: J Oncol. 2020 Jul 15;2020:3417036. doi: 10.1155/2020/3417036 (PMC7378602; doi:10.1155/2020/3417036)
Supplement: Supplementary Materials — Figure S1: sensitivity analysis and publication bias. (a) Sensitivity analysis of the association between SNHG15 expression and association and recurrence-free survival (RFS). (b) Begg's funnel plot of publication bias for RFS. [file 3417036.f1.pdf]

# Supplementary Materials

a

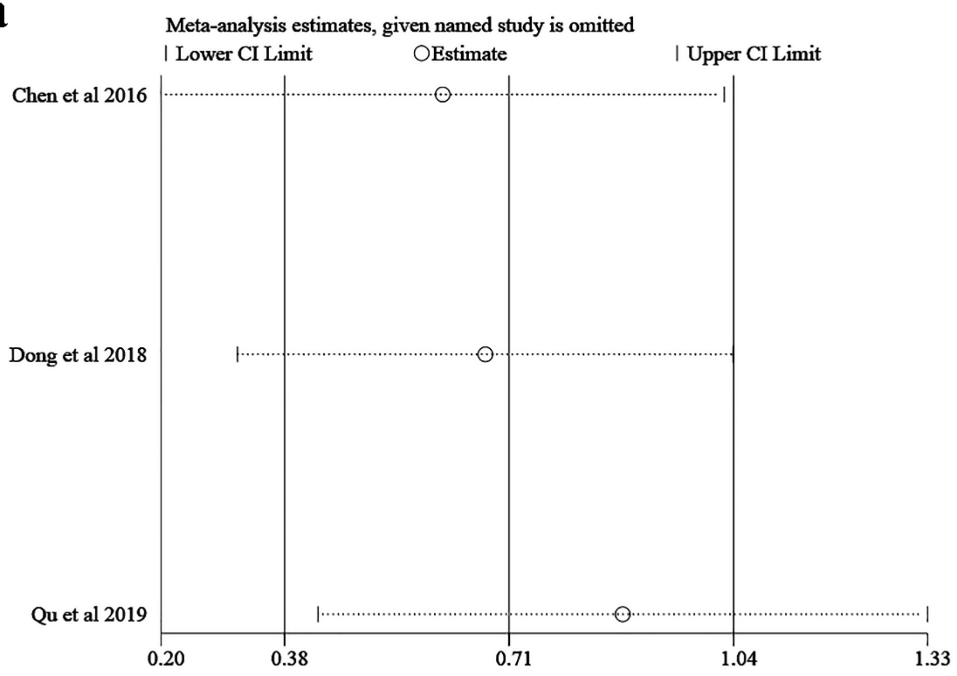


b

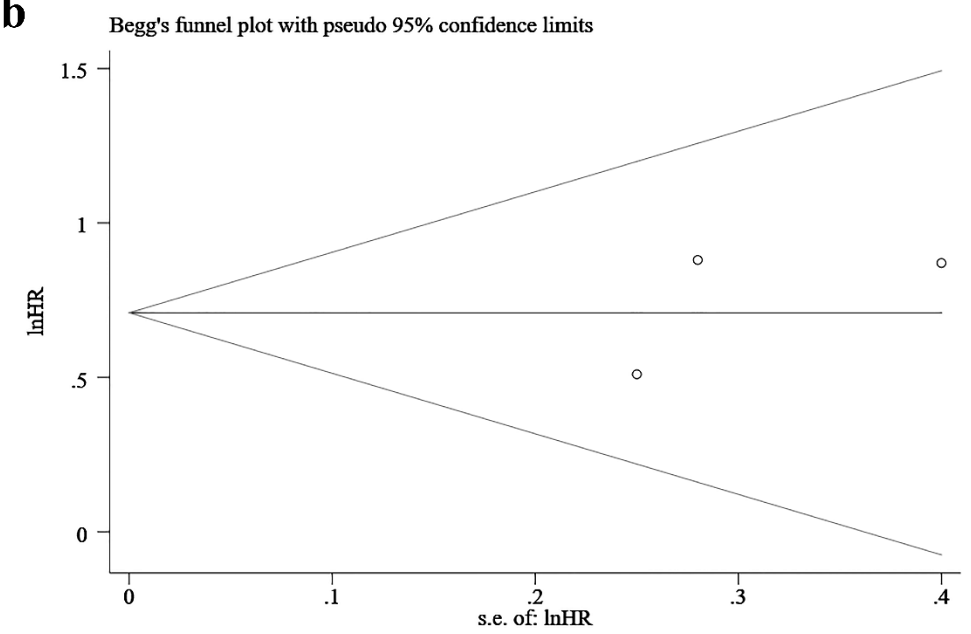


Figure S1: Sensitivity analysis and publication bias. (a) Sensitivity analysis of the association between SNHG15 expression and recurrence-free survival (RFS). (b) Begg's funnel plot of publication bias for RFS.

# CERTIFICATE OF ENGLISH EDITING

This document certifies that the paper listed below has been edited to ensure that the language is clear and free of errors. The logical presentation of ideas and the structure of the paper were also checked during the editing process. The edit was performed by professional editors at Editage, a division of Cactus Communications. The intent of the author's message was not altered in any way during the editing process. The quality of the edit has been guaranteed, with the assumption that our suggested changes have been accepted and have not been further altered without the knowledge of our editors.

## Title Of The Paper

High expression of the long non-coding RNA SNHG15 in cancer tissues samples predicts an unfavorable prognosis of cancer patients: A meta-analysis

## Authors

Cheng Zhang, Yang Ke, Xin Liu, Xinhong Wang, Yuehua Li, Jian Zhou, Heng Zhang, Lin Wang

## Job Code

IFQAY_1

editage by CACTUS

Signature

Vikas Narang

Vikas Narang,
Chief Operating Officer,
Editage

Date of Issue
**May 21, 2020**

Editage, a brand of Cactus Communications, offers professional English language editing and publication support services to authors engaged in over 500 areas of research. Through its community of experienced editors, which includes doctors, engineers, published scientists, and researchers with peer review experience, Editage has successfully helped authors get published in internationally reputed journals. Authors who work with Editage are guaranteed excellent language quality and timely delivery.

INFORMATION SECURITY MANAGEMENT SYSTEM DNV·GL ISO/IEC 27001

CACTUS.

**Contact Editage**

| Worldwide | Japan | Korea | China | Brazil |
| --- | --- | --- | --- | --- |
| request@editage.com | submissions@editage.com | submit-korea@editage.com | fabiao@editage.cn | contato@editage.com |
| +1 877-334-8243 | +81 03-6868-3348 | 1544-9241 | 400-005-6055 | 0800-892-20-97 |
| www.editage.com | www.editage.jp | www.editage.co.kr | www.editage.cn | www.editage.com.br |
